# Uptake and Presence Evaluation of Nanoparticles in *Cicer arietinum* L. by Infrared Spectroscopy and Machine Learning Techniques

**DOI:** 10.3390/plants11121569

**Published:** 2022-06-14

**Authors:** Feyza Candan, Yuriy Markushin, Gulnihal Ozbay

**Affiliations:** 1 Department of Biology, Faculty of Science and Letters, Manisa Celal Bayar University, 45140 Manisa, Turkey; feyza.candan@cbu.edu.tr; 2 Division of Physics, Engineering, Mathematics, and Computer Science, College of Agriculture, Science and Technology, Delaware State University, Dover, DE 19901, USA; ymarkushin@desu.edu; 3 Department of Agriculture and Natural Resources, College of Agriculture, Science and Technology, Delaware State University, Dover, DE 19901, USA

**Keywords:** *Cicer arietinum* L., gold nanoparticles, carbon nanotubes, ATR-FTIR spectroscopy, machine learning techniques, principal component analysis, support vector machine classification

## Abstract

The aim of this work was to study the applicability of infrared spectroscopy combined with machine learning techniques to evaluate the uptake and distribution of gold nanoparticles (AuNPs) and single-walled carbon nanotubes (CNTs) in *Cicer arietinum* L. (chickpea). Obtained spectral data revealed that the uptake of AuNPs and CNTs by the *C. arietinum* seedlings’ root resulted in the accumulation of AuNPs and CNTs at stem and leaf parts, which consequently led to the heterogeneous distribution of nanoparticles. principal component analysis and support vector machine classification were applied to assess its usefulness for evaluating the results obtained using the attenuated total reflectance-Fourier transform infrared spectroscopy method of *C. arietinum* plant grown at different conditions. Specific wavenumbers that could classify the different nanoparticle constituents of *C. arietinum* plant extracts according to their ATR-FTIR spectra were identified within three specific regions: 450–503 cm^−1^, 750–870 cm^−1^, and 1022–1218 cm^−1^, based on larger PCA loadings of *C. arietinum* ATR-FTIR spectra with distinct spectral differences between samples of interest. The current work paves a path to the future fabrication strategies for AuNPs and single-walled CNTs via plant-based routes and highlights the diversity of the applications of these materials in bio-nanotechnology. These results indicate the importance of family-plant selection, choice of methods, and pathways for the efficient biomolecule delivery, drug cargo, and optimal conditions in the wide spectrum of bioapplications.

## 1. Introduction

Effects of nanoparticles (NPs) on biological systems and for the environment are known according to recent studies [1,2,3,4,5]. Plants are one of the most important organisms of our ecosystem and they also face toxicity caused by contamination of NPs in the soil. Thus, it is necessary to understand the interactions of NPs with plants, essential base components of all ecosystems [6,7,8]. On the other hand, as NPs are being increasingly used in many sectors of the industry, there is growing interest in the biological and environmental safety of their production by using plant extracts as a model system [9]. Considerably, studying all of these NPs-related topics will face challenges without having an efficient, robust, and cost-effective system to differentiate the NPs composition, and to identify the uptake and presence of SNPs inside the plants.

In the 21st century, many investigations have been related to the economic plants uptake, distribution, translocation, and accumulation of NPs, for example: silver NPs (SNPs) in *Brassica juncea* and *Medicago sativa* [10], ceria NPs in cucumber [11], Au NPs in rice, radish, pumpkin, and perennial ryegrass [12], SNPs in wheat [13], Au NPs and SNPs in rice [14,15,16], AuNPs and CNTs in pea green [17,18], and SNPs in tomato [19].

*Cicer arietinum* L. is an annual plant from the Fabaceae family and it is one of the first plants cultivated in the world. Molecular analysis demonstrates that the *C. arietinum* is from the southeastern Anatolia region of Turkey, and the first information about the *C. arietinum* wild species in the world is documented from the Hacılar village in Turkey [20]. In this region, *C. arietinum* has been grown for about 7000–7500 years [21,22,23].

Because of its economic value (16.2 billion USD global trade in 2020) and its easy adaptive conditions for laboratory, *C. arietinum* was selected as a plant model to observe the absorption potential into the seeds and transportation-translocation of AuNPs and single-walled CNTs as regards the root, stem, and leaf of the *C. arietinum* seedlings. The spectral differences between the control group and Au NPs- or CNTs-exposed *C. arietinum* seeds obtained with ATR-FTIR were studied by employing machine learning techniques. Data obtained from the Au NPs- and CNTs-exposed seedlings and from the control samples were compared and evaluated according to Au NPs and CNTs concentration distribution-accumulation on the plant organs and their comparative significant importance, thus its general value in other perspectives.

Attenuated total reflectance-Fourier transform infrared spectroscopy (ATR-FTIR) has become an attractive analytical method because it can be used with a little or essentially no sample preparation, and analysis is relatively rapid [24]. These advantages and the small sample volume result in time and cost savings, which consequently lead to an increase in the number of analyzed samples. That is important for the future in-field applications.

Support vector machine (SVM) is a pattern recognition and classification method that is used widely in data mining applications for the purpose of supervised classification of data representing different classes of interest [25]. SVM is based on statistical learning to determine a hyperplane for optimal separation of classes. SVM uses an iterative training algorithm to achieve separation of different classes.

Principal component analysis (PCA) is typically used to provide a visual representation of the relationships between samples and variables and to combine samples into classes [26]. In this work, PCA was applied especially for the translation of spectroscopic data into lower dimensional space, and PCA score plots were used to objectively classify extract samples of *C. arietinum* plant, whereas SVM analysis allowed for the classification of the plant root, stem, and leaf extract samples based on the similarities of their spectroscopic properties (due to NPs components). The hypothesis of the study was to differentiate the NPs composition of the plant samples using *C. arietinum* as a model based on its spectroscopical data.

## 2. Materials and Methods

### 2.1. Seedling Growth and Extract Preparation

In our study, the water soluble single-walled CNTs functionalized with polyethylene glycol (PEG) obtained from Carbon Solutions at the concentration of 1.25 mg/mL in sterile distilled water was used for the seed-NPs exposure. We also used 10 nm of diameter Au NPs (optical density (OD) 1, stabilized suspension in 0.1 mM phosphate-buffered saline (PBS), reactant free). The Au NPs was obtained from Millipore Sigma and subsequently, a 4x-dilution with sterile distilled water was used for the seed-NPs exposure.

The following are CNT properties obtained from [27]: type of material P7-CNTs; individual tube lengths range from 0.5 to 3 µm and have an average diameter of 1.4 nm. CNTs tend to occur as bundles with bundle lengths of 1–5 µm and average bundle diameters of 2–10 nm and bundle length: 500–600 nm; bundle diameter: 4–5 nm [27]. The same commercial CNTs that we used in our work were characterized in [28]. In this work P7-CNTs were obtained from commercial P3-CNTs by derivatizing with PEG to give water solubility. According to [28], the characterization of P7-CNTs revealed that the zeta potential of CNTs in a pH range of the soil used in our work (from pH 6 to pH 8) is about—50 mV.

The following are 10 nm diameter gold nanoparticles properties obtained from [28]: polydispersity index (PDI) ≤ 0.2, core size: 8–12 nm, hydrodynamic diameter (Z): 11–25 nm; concentration of particles/mL: 5.38 × 10^12^–6.58 × 10^12^, absorption max: 510–525 nm, OD 1, zeta potential of −25.8 mV at pH 7.4 in stabilized suspension in 0.1 mM PBS (reactant free) that we used in our work [29].

In the next step, 18 *C. arietinum* seeds (*cv.* dried raw garbanzo beans) were sterilized with sodium hypochlorite (7.5%) for 20 min. Subsequently, seeds were rinsed with autoclaved-distilled water 3 times and seeds were taken to sterile tubes for further analysis [30]. Later, 6 seeds were treated for duration of 2 days with 15 mL Au NPs (1.25 mg mL^−1^, 10 nm of diameter): water (group I). In parallel, 6 other *C. arietinum* seeds were treated with 15 mL CNTs (OD: 0.25): water for the duration of 2 days (group II); and 6 other *C. arietinum* seeds were used as control (group III) and seeds were treated with 15 mL pure water for duration of 2 days.

After 2 days, all three groups of seeds were planted into 0.5 L pots (10.5 cm × 9 cm) for 3 weeks in growth chamber (22 ± 24 °C, humidity = 60%, 10-h light period, intensity: 250 μmol/m^2^/s). The residual water (group III) and two NPs solutions (group I and II) which remained in each tube after 2 days seeds NPs treated cultures were poured directly onto the seeds during planting process. All the groups were checked every 24 h and each pot was irrigated with 8 mL distilled water.

After 3 weeks, the control, Au NPs, and CNTs groups were photographed, and different parts of the *C. arietinum* plants (root, stem, and leaf) were sampled, and homogenized and washed by deionized water. Afterward, the samples were collected for centrifugal filtration, and for agitation, the plant samples were vortexed (10 s) and centrifuged for 30 min at the speed of 13,000 rpm (24 °C).

### 2.2. Data Collection and Analysis

For analysis of the ATR-FTIR spectral data, we used the multivariate data analysis and machine learning techniques using PCA and SVM. For this purpose, we utilized the Unscrambler software (CAMO Analytics). The SVM method was applied with the following parameters: SVM type: Classification (nu-SVC), Kernel type: Radial basis function, Gamma: 0.0005353319, Nu value: 0.5, Weights: All1.00, Cross validation segments: 10. We used 26 *C. arietinum* plant samples for multivariate data analysis, with 1868 variables representing ATR-FTIR spectral wavenumbers for the 400–4000 cm^−1^ spectral range and 572 wavenumbers for the specific range from 400 to 1500 cm^−1^. The cross-validation procedure involved taking the training set and splitting it into 10 segments in a random way and then to be trained with the SVM algorithm on 9 parts to test on the 10th part.

In this study, the SVM classification method was used based on our previous comparisons among other classification methods such as K-nearest neighbor, classification and regression trees, neural networks, SVM, adaptive local hyperplane, and linear discriminant classifiers for spectroscopic data sets. Our previous studies strongly show that SVM is one of the most robust and accurate algorithms for spectroscopic data sets [24,31]. To minimize a very strong IR absorption of water in several regions related to its O–H stretching and different bending vibrations [32], we used dried samples for ATR-FTIR analysis. Nevertheless, some residual water might still be present in dried samples. It is well known that the general regions of the infrared spectrum in which various kinds of vibrational bands have been observed are associated to water molecule (i.e., ~1595, and ~3657 cm^−1^) [32]. Therefore, the spectra were collected over the range 400–1500 cm^−1^ to minimize the potential influence of several regions related to O–H stretching and different bending vibrations of water molecule.

Finally, 5 μL aliquots from each tested group were placed on the diamond crystal plate of the spectrometer and dried (room temperature for 30 min). The dried samples were subsequently analyzed by the ATR-FTIR (Nicolet 6700 ATR-FTIR Spectrometer from Thermo Electron Corporation, Waltham, MA, USA). Drops of the plant extract samples were deposited over an aperture on the top of the device, and the aperture was connected to the surface of a diamond prism where the total reflection occurs. The ATR-FTIR spectra are collected with a resolution of 4 cm^−1^. A total of 100 scans were averaged for each spectrum. The background for the ATR-FTIR spectra is a spectrum of empty ATR crystal in the air.

## 3. Results

### 3.1. Morphological Results

The physical interactions related to the Au NPs and CNTs in the water occur via passing the seed coat and semipermeable cell walls with the pure water. Since the seeds had no endosperm at the maturity stages in *C. arietinum*, two developed cotyledons interfaced with the NPs. Accordingly, the embryo of the seeds which were treated for 2 days with Au NPs and CNTs solutions through their development process had cotyledons, which gave the nutrients to the plumula (which gave nutrients to the stem), radicula (which gave nutrients to the root), and hipocotyl (which gave nutrients to the part between root and stem). These embryos interfaced the two tested NPs via the swollen cotyledons processes, and later, the NPs interfered with other parts of the plant through transport and development processes.

**Figure 1:**
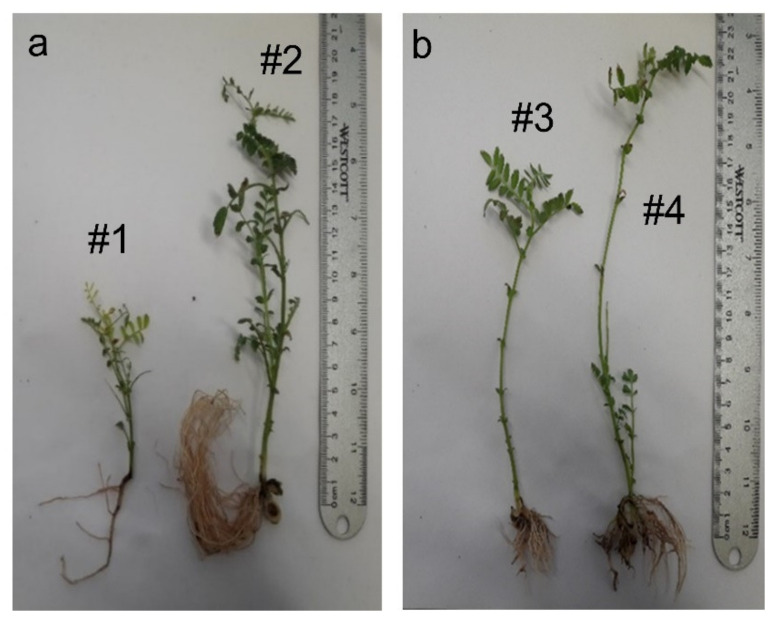
Photos of typical *C. arietinum* seedling of the control group (#1 in frame (**a**)) and the sample treated with: Au NPs (#2 in frame (**a**)) and with CNTs (#3 and #4 in frame (**b**)) (Pictures taken by F. Candan).

Bioimages revealed that our tested groups had different morphologies (Figure 1a,b), and the stem was growing more vertically and branched in our control plants (4.8–5.5 cm).

We also found that, among the tested groups, the highest stems (height: 14.8–26.2 cm) were recorded for the CNTs, and the most leaves and lateral branching were observed on stems with Au NPs (height: 15.2–17.8 cm) (Figure 1). In all three analyzed groups, the leaves had alternate arrangement, with an imparipinnate compound leaf shape, and serrate edges. We did not observe any morphological changes on the leaflets (foliole) in Au NPs and CNTs groups compared to the control. However, in all the AuNPs and CNTs samples, the imparipinnate leaves’ number, size, and leaflets were increased in length and width, and leaflet colors were observed in dark green (Figure 1). The study of the root system in our analyzed groups revealed that even though the *C. arietinum* in our control group had a taproot rooting system consisting of primary root (0.9–4.6 cm) (Figure 1a (#1)) the Au NPs and CNTs groups had longer and more lateral roots. The lengths of the root were recorded as 5.3–7.2 cm and 1.8–3.9 cm, respectively, for Au NPs and CNTs.

The efficiency of the carbon nanoparticles (CNPs) was also studied on the morphology and physiology of *Vigna radiate* from the Fabaceae family. The results revealed the CNPs (100 to 150 µM) had a positive influence on the growth of the *V. radiate* and the plant biomass (fresh weight) increased 1.12-fold, total concentration and protein content also increased 1.9- and 1.14-fold, respectively [33].

### 3.2. Spectroscopic Results

**Figure 2:**
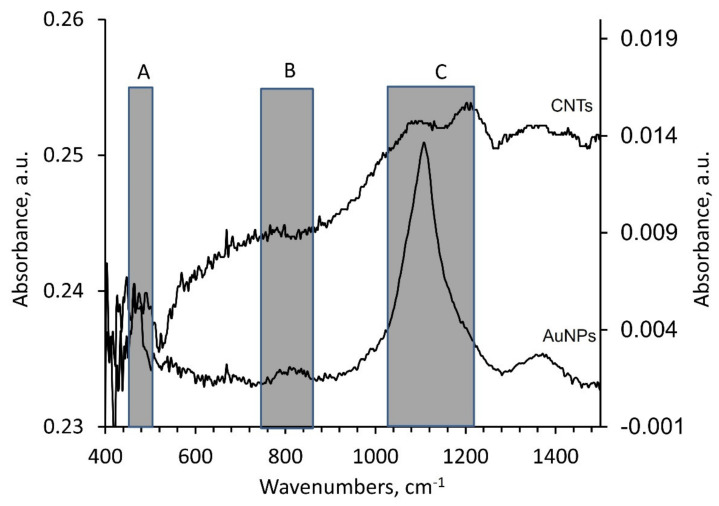
ATR-FTIR spectra of gold nanoparticles (AuNPs, right *Y*-axis) and carbon nanotube samples (CNTs, left *Y*-axis). Marked spectral ranges: (A) 450–503 cm^−1^, (B) 750–870 cm^−1^, (C) 1022–1218 cm^−1^.

**Figure 3:**
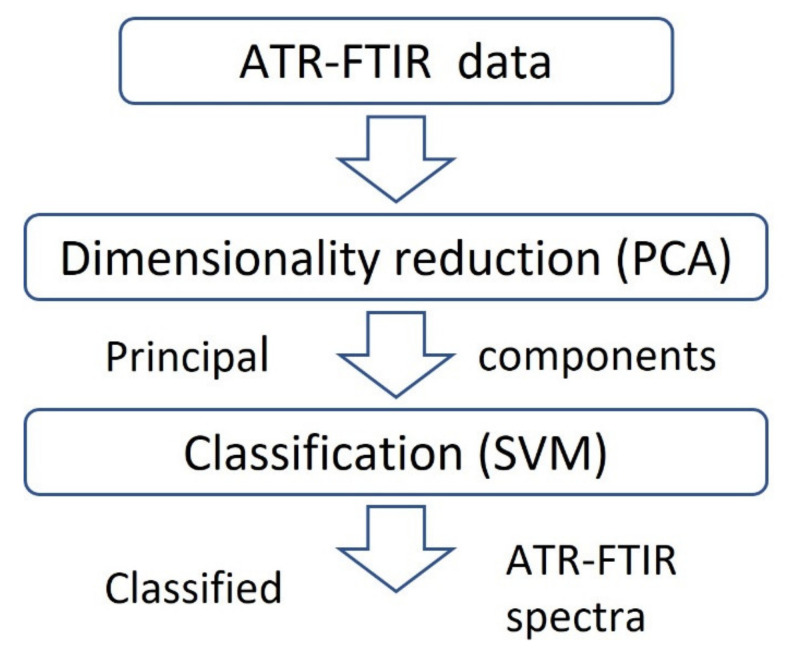
Statistical framework for automatic ATR-FTIR spectra classification (adapted from [24]).

**Figure 4:**
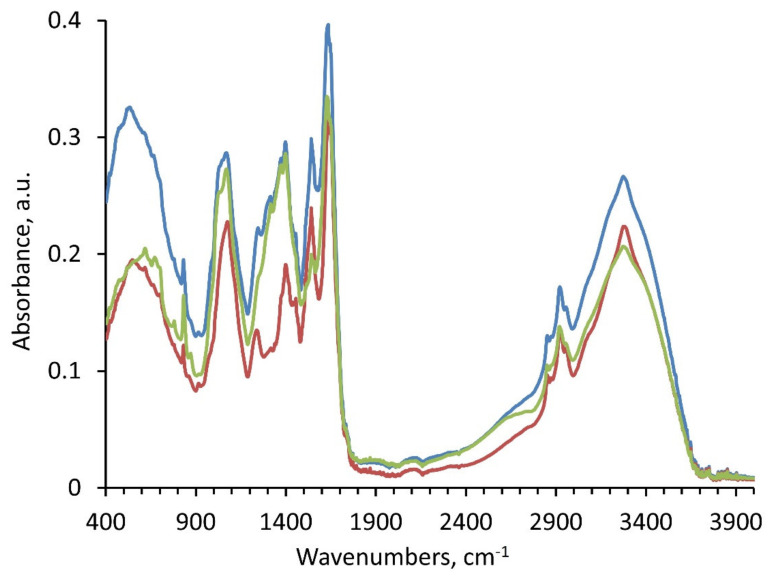
ATR-FTIR spectra of *C. arietinum* plant samples grown under the influence of Au NPs and CNTs. Note: Control—red line, Au NPs-treated plant—blue line, and CNTs-treated plants—green line. The ATR-FTIR clearly identified three tested groups.

**Figure 5:**
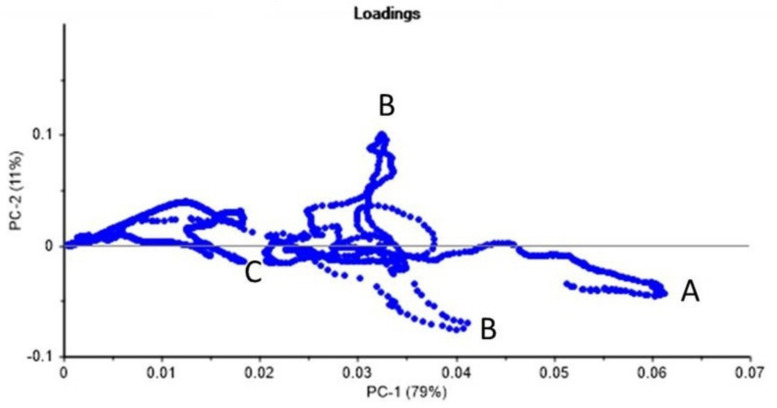
PCA loading ATR-FTIR spectral ranges (A, B, C) for *C. arietinum* root samples grown at three conditions (Class). See text and Table 1 for more details.

**Figure 6:**
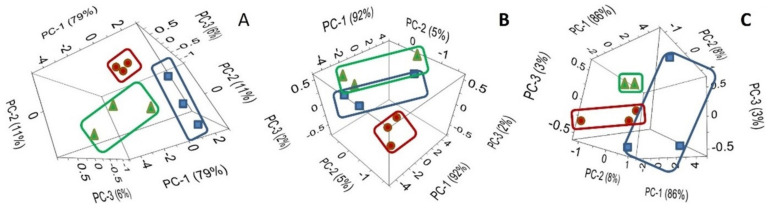
The PCA score graph of the first three PC for ATR-FTIR spectral data of *C. arietinum* root (**A**), stem (**B**), and leaf (**C**) samples grown in the presence of: (1) Au NPs (blue), (2) CNTs (red), and (3) control group (green).

It is documented that the NPs can be transported to the above-ground portion of the plants and to the shoots and leaves through the plant vascular systems [34]. The Au NPs and CNTs possible transportation and translocation from the roots to stem and ultimately to the leaves by vascular system was evaluated by analyzing the spectral data in our study (Figure 2 and Figure 3). These data are presented in tables (Table 1, Table 2 and Table 3) and graphs (Figure 2, Figure 4, Figure 5 and Figure 6). For this purpose, the ATR-FTIR results from the root, stem, and leaves in all of our tested groups were collected, and consequently the PCA-SVM technique was used to classify samples based on the spectral differences due to the presence of AuNPs or CNTs.

Previously, we reported an efficient statistical framework for automatic classification of the ATR-FTIR spectra of various proteins which potentially can be used as biomarkers of ovarian cancer: monoclonal antibodies and antigens of ovarian cancer marker CA125, Osteopontin, Leptin, and insulin-like growth factor II [24]. We also applied this efficient established method in our lab for the classification of different plant extract samples (Figure 3). Through this framework, we follow several steps as follows: (1) dimensionality reduction (the number of wavenumbers in ATR-FTIR spectra is reduced using PCA method), (2) the obtained principal components values are used as an input for the classification of ATR-FTIR spectra.

PCA is a commonly used dimensionality reduction method [26]. By this method, the PCA analysis reduces the dimensionality of a dataset consisting of multiple interrelated variables and retaining of the variation present in the dataset. PCA creates the new variables by transforming the original variables in a dataset to a new set of variables, called the principal components (PC). The first PC typically accounts for the maximal variation of data.

We propose the identification of specific ATR-FTIR wavenumbers that could classify samples based on inclusion of AuNPs or CNTs from the aqueous extracts of *C. arietinum* grown under the influence of AuNPs or CNTs, based on the collection of ATR-FTIR spectra within three specific spectral ranges as follow, (A) 450–503 cm^−1^, (B) 750–870 cm^−1^, and (C) 1022–1218 cm^−1^ (Figure 2). These spectral ranges were selected based on distinct spectral differences between the ATR-FTIR spectra of Au NPs and CNTs samples, which are important for the classification of the plant samples treated by NPs (Figure 2).

Figure 5 demonstrates the PCA loading plot in order to identify the variables (wavenumbers) with the largest effect on the studied NPs. In this regard, larger PCA loadings indicate that the variable strongly influences the component, and the PCA loadings close to 0 indicate the variable has a weak influence on the component. Table 1 shows the comparison of spectral ranges for PCA loading with stronger effect on the PC for the following classes of samples: (1) root samples of *C. arietinum* plant grown at three conditions (column 1 in Table 1) and (2) the specific ATR-FTIR spectral peaks/valleys of AuNPs- (column 2 in Table 1) and CNTs-standards (column 3 in Table 1).

The variable PCA loadings for PC-1 and PC-2 presented in Figure 5 show peaks A, B, and C related to the following spectral ranges in Table 1 and in Figure 2: (1) range A at 450–503 cm^−1^; (2) range B at 750–870 cm^−1^; and (3) range C at 1022–1218 cm^−1^. We used these identified spectral ranges A, B, and C for the dimensionality reduction by PCA analysis and for the classification of the NPs-treated plant samples using the SVM method.

Since the main purpose of the PCA is the dimensionality reduction of the spectral dataset, the purpose of using the SVM classification is to compute a separation hyperplane for optimal separation of the spectral data assigned to different classes, to maximize the minimal distance between points and the separation hyperplane [24,25]. Such constructed hyperplane provides the best generalization of unknown examples. SVM utilizes the structural risk minimization principle with the goal to achieve zero training error while minimizing the complexity of the model [24,25].

PC analysis has also been applied for the feature extraction of ATR-FTIR spectral data to visually demonstrate class separability (Figure 5). Note that dimensionality reduction is essential in classification [31]. The number of attributes can be large (e.g., 1868 variables representing ATR-FTIR spectral wavenumbers for the 400–4000 cm^−1^ spectral range and 572 wavenumbers for the specific range from 400 to 1500 cm^−1^). It is also known that not all the attributes available to a learning algorithm are useful [35].

In our study, we used the first two or three PCs for classification of experimental data, mostly because of our daily experience in inhabiting a space of three dimensions. Therefore, when researchers visually analyze the three-dimensional data (Figure 3), they implicitly perform relevant discrimination leading to really good classification results with the visual inspection.

Figure 6 represents the PCA score graph of the first three PC for the ATR-FTIR spectral data of *C. arietinum*. Our results clearly demonstrated that even the first three PCs are sufficient to achieve separation of 2 NPs-based and 1 control group classes (blue squares for Au NPs, red circles for CNTs, and green triangles for control group) for ATR-FTIR spectral data of *C. arietinum* root (A), stem (B), and leaf (C) samples.

The first principal component in Figure 6A explains 79% of the variability, the second PC explains 11%, and the third only 6% of variability. Together, the first three PCs explain 96% of the variability. PC-1 in Figure 6B explains 92% of the variability, PC-2 explains 5%, and PC-3—only 2%. Together, the first three PCs explain 99% of the variability. At the same time, PC-1 in Figure 6C explains 86% of the variability, PC-2 explains 8%, and PC-3—only 3%. Together, the first three PCs explain 97% of the variability. As the explained variability values are close to 100% (e.g., 96%, 99%, and 97%), and in order to minimize the possible overfitting, the cross-validation for the SVM classification was performed.

Table 2 includes SVM classification for ATR-FTIR spectral data of *C. arietinum* samples (all plant parts combined for the analysis) grown at three conditions (Class): (1) Au NPs (CP Au NPs), (2) CNTs (CP CNTs), and (3) control ground (CP control). Correct SVM prediction is marked by the bold green fonts (green is correct prediction and red is wrong prediction). The SVM prediction matrix presented in Table 2 indicates the classification determined for each plant sample. From a total of 26 samples analyzed, 22 samples had correct SVM prediction of the class and 4 had incorrect SVM prediction of the class with total SVM prediction accuracy of about 85%. Therefore, the application of SVM is able to provide about 85% prediction accuracy on the *C. arietinum* samples grown at three conditions with all the plant parts combined for the analysis.

Table 3 presents the training and cross-validation accuracy of the SVM classification for ATR-FTIR spectral data of various plant parts of the *C. arietinum* samples grown at three conditions: (1) Au NPs, (2) CNTs, and (3) control ground. In this regard, Table 3 shows: 1st table row-data related to all plant parts analyzed; 2nd table row-data related to the plant root samples; and 3rd table row-data related to the plant stem samples.

The comparison of the cross-validation accuracy values for the root samples (~78%), the stem (~44%), and for the leaves (~33%) shows that the accuracy of the proposed model for the unknown samples was in good agreement with the possible translocation and accumulation pathway of nano-inclusions inside the plant structure (from root-to stem-to leaves). Larger cross-validation accuracy value for root samples is likely associated with larger concentration of Au NPs and CNTs in the root-extracts of *C. arietinum* plant grown at different conditions, as compared with the stem- or leaf-extracts.

## 4. Discussion

In our study, the obtained spectral data were in agreement with heterogeneous distribution of AuNPs and CNTs in *C. arietinum* seedlings’ root, stem, and leaf (Figure 6, Table 3). Moreover, as it is clearly demonstrated in Figure 6, in the analysis of the PCA scores of the first three PCs for the ATR-FTIR spectral data of *C. arietinum*, even the first three PCs are sufficient to achieve the visual separation (clustering) of 2 NPs-based and 1 control group classes for ATR-FTIR spectral data of *C. arietinum* root, stem, and leaf samples (Figure 5 and Figure 6).

By comparing the spectra of the Au NPs and CNTs samples (Figure 2), we identified three specific spectral ranges, 450–503 cm^−1^, 750–870 cm^−1^, and 1022–1218 cm^−1^, to be used for the dimensionality reduction by PCA and for the classification by SVM of the aqueous extracts of *C. arietinum* used as a model plant grown under influence of Au NPs and CNTs. Those spectral ranges were chosen based on larger PCA loadings of *C. arietinum* ATR-FTIR spectral ranges (Figure 5, Table 1), which also overlap with spectral ranges with distinct visual differences between spectra of Au NPs and CNTs samples (Figure 2). We hypothesize that the *C. arietinum* root samples demonstrate stronger PCA class separability than either the leaf or the stem samples, due to the most probable route of nanoparticular transportation in plants. We also observed that the Au NPs and CNTs appeared in the spectral study differently (Figure 6B,C and Table 3). These differences might be related to possible variance of the chemical bonds created between NPs and the plant components or to photocatalytic effects or due to possible promotion of higher photosynthetic activity in the *C. arietinum* by CNTs [36].

Previously, it was shown in the AuNPs-exposed barley plants that the AuNPs were accumulated in the plants root up to a certain extent and it permanently inhibited the root growth [16]. Based on our findings, the Au NPs- and CNTs-exposure of *C. arietinum* seeds at our used concentration leads to an increase in the length of roots, stems, and leaves in *C. arietinum* Plant (Figure 1). The spectral results are in agreement with the hypothesis of more significant translocal distribution of AuNPs and CNTs in the root system of the plant than the leaf or the stem parts (Figure 6, Table 3). The difference of our results from the previous reported work (16] could be explained by the difference of the Au NPs size used in our study (10 nm of diameter), by the specific type of the plant family (Poaceae-Fabaceae), or based on the specific interactions of the monocotyl-dicotyl plants with our tested NPs.

Previously, in the work [37], different sizes of Au NPs (6–100 nm of diameter) were synthesized from *Lantana camara* (Verbenaceae family) leaf extracts by various methods and ATR-FTIR studies were done as well. Therefore, this plant was recommended for various medicinal and biomedical applications [37]. The ATR-FTIR results in our study also showed permissible results for our tested NPs (Figure 2, Figure 5 and Figure 6). At the same time, the Fabaceae (Leguminosae) family taxa is more common, it is easier to grow, and it has greater economic value when compared with the Verbenaceae family. Therefore, this type of plant can be recommended for nanotechnological applications.

It is known that cucumber seedlings treated with 7 nm of diameter ceria particles showed significantly higher ceria contents in both roots and shoots than those exposed to 25 nm of diameter ceria particles at all test concentrations. Only very limited amounts of ceria nanoparticles could be transferred from the roots to shoots because the entry of nanoparticles into the roots was difficult [11]. As is seen, diameters of NPs and nanomaterials transport differentiation change from plant to plant. Because of that, our study provides valuable information about *C. arietinum*-Au NPs (10 nm of diameter) harmony and *C. arietinum*-CNTs harmony, which are both remarkable for other fields of study, especially for biomedical-based ones (Figure 5 and Figure 6, Table 1, Table 2 and Table 3).

Effects of Au NPs (from 10 to 14 nm of diameter) on leaves and chloroplasts have been also studied with the conclusion that photosynthetic capacity is greater in the presence of Au NPs [38]. On the other hand, it was also demonstrated that CNTs are capable of developing the chloroplast carbon capture and photosynthesis by improving the chloroplasts activities [39]. According to our study, even with a naked eye, leaves of seedlings treated with Au NPs and CNTs being dark green is the result of an increase in chloroplast caused by physiologic activities in the plant (Figure 1) [5,40]. In addition to that, the detailed stereo microscopic analyses of the leaves also showed the same result. However, detailed comparative physiological studies are needed on this subject.

Cano et al. [41] studied CNTs and the effects on corn (*Zea mays* L.) relative to uptake, accumulation, and stress features. As a result, they reported that CNTs were taken up into corn roots, stems, and leaves, and that CNTs accumulated mostly in roots, with minimal accumulation in stems and leaves. All these results are in very good agreement with our study (Figure 6, Table 3), despite the different methods employed in both studies.

On the other hand, root nodules appeared as a result of root trichomes and soil bacterium synergy in Fabaceae family and then the nitrogen fixation could be provided to soil [42,43]. The increase of the lateral roots and correlatedly with the *Rhizobium* bacteria means that the particle concentration was shown to enhance all yields on the soil and under the soil development in a more effective way than lower concentrations of the CNTs or of the multiwalled CNTs. According to our study, increasing in lateral roots can be clearly seen in Figure 1. This information highlights the possible structural adaptation and correlations between *C. arietinum* and the microbial biota increasing by adding CNTs. On the other hand, our study shows the possibility of the CNTs application directly to seeds water solution, not to soil, which is cheaper and easier to implement in practice.

The limits of uptake of the metallic silver by two common metallophytes, *Brassica juncea* and *Medicago sativa*, and assessing of the form and distribution of the SNPs by the plants was also studied in [10]. According to this study, *M. sativa* belonging to Fabaceae family translocated more SNPs than *B. juncea* which belongs to Brassicaceae family [10]. *Medicago sativa* and *C. arietinum* plants showed a preference trend to SNPs and Au NPs and they both belong to the Fabaceae family. Thus, we recommend the *C. arietinum* as a potential metallophyte model in future studies (Table 1, Table 2 and Table 3, Figure 1, Figure 2, Figure 5 and Figure 6).

The role of the Au NPs and CNTs in *C. arietinum* plant signal transduction between cells of the roots, stems, and leaves and developmental differences, especially in their physiology metabolism, can be studied further in light of the current investigation. However, this work showed that *C. arietinum* seeds are potentially capable of absorbing Au NPs and CNTs, with possible transfer and translocation pathways as it can be seen from the plant morphology and spectral graphs (Figure 1, Figure 2, Figure 5 and Figure 6).

## 5. Conclusions

The present study relates to the applicability evaluation of infrared spectroscopy combined with machine learning techniques for monitoring the uptake and distribution of Au NPs and CNTs in *C. arietinum* samples. The results indicate that the principal component analysis of the infrared spectroscopic data leads to good classification results with the visual inspection. The obtained results further demonstrate the possibility of automatic classification of plant parts based on NPs-inclusion in plant samples using PCA and linear SVM with an accuracy of nearly 85%. It was also shown that application of the ATR-FTIR for NPs-inclusion in plant samples can potentially lead to the development of future analytical techniques and applications.

Additionally, the obtained results might be helpful in evaluating plants, especially economically valuable plants as an important component of the ecological systems and need to be considered when developing possible transportation and accumulation pathways of nanomaterials from the environment to the human body.

The results of the current study showed that rapid-growing plants such as Fabaceae family members such as *C. arietinum* might be useful in environmental remediation, phytoremediation, and phyto-mining, since our study showed the transfer and translocation of NPs from the root system to the upper part (stem and leaves) of *C. arietinum.* Because of that, we recommend more studies on NPs combined with *C. arietinum* in other fields such as biomedical studies.

Moreover, the plant morphogenesis and differentiation are formations that are complementary to each other but essentially do not control each other, as their formations are controlled by different genes or gene complexes. Therefore, it is thought that because of NPs accumulation observed in seedlings treated with Au NPs and CNTs, there might be remarkable change in the genes which control plant growth and differentiation. Consequently, we plan to carry out a complementary study to evaluate the *C. arietinum* samples of plants treated with Au NPs and CNTs by comparing them with the control group in terms of genetics.

## Figures and Tables

**Table 1:** Comparison of spectral ranges for PCA loading with stronger effect on the PC of the *C. arietinum* root samples grown under influence of NPs with the ATR-FTIR spectral peaks/valleys of AuNPs and CNTs standard samples. (A): Range A at 450–503 cm^−1^; (B): range B at 750–870 cm^−1^; and (C): range C at 1022–1218 cm^−1^.

Spectral Ranges for PCA Loading with Stronger Effect on the PC for *C. arietinum* Root Samples Grown under Influence of NPs, (cm^−1^)	ATR-FTIR Peaks, AuNPs-Standard, (cm^−1^)	ATR-FTIR Peaks/Valleys CNTs-Standard, (cm^−1^)
450–503 (A)	450–503	490–560
750–870 (B)	750–870	790–850
1022–1218 (C)	1022–1218	1130–1260

**Table 2:** SVM classification for ATR-FTIR spectral data of *C. arietinum* samples (all plant parts combined for the analysis) grown at 3 conditions (Class): (1) Au NPs (Au NPs), (2) carbon nanotubes (CNTs) and (3) control ground (control). Correct SVM prediction is marked by the bold green font (green is correct prediction and red is wrong prediction).

Samples	Class	SVM Prediction
CP leaf1	Au NPs	Au NPs
CP root1	Au NPs	Au NPs
CP stem1	Au NPs	Au NPs
CP leaf1	CNTs	CNTs
CP root1	CNTs	CNTs
CP stem1	CNTs	CNTs
CP root1	control	control
CP stem1	control	Au NPs
CP leaf2	Au NPs	Au NPs
CP root2	Au NPs	Au NPs
CP stem2	Au NPs	control
CP leaf2	CNTs	control
CP root2	CNTs	CNTs
CP stem2	CNTs	CNTs
CP leaf2	control	control
CP root2	control	control
CP stem2	control	control
CP leaf3	Au NPs	Au NPs
CP root3	Au NPs	Au NPs
CP stem3	Au NPs	Au NPs
CP leaf3	CNTs	Au NPs
CP root3	CNTs	CNTs
CP stem3	CNTs	CNTs
CP leaf3	control	control
CP root3	control	control
CP stem3	control	control

Note: In the current study for the ATR-FTIR measurements, we used 9 plants grown in 3 different conditions (3 plants per each condition). From each plant, we collected 3 types of samples: leaf, steam, and root. The total is 27 samples. One control leaf sample was lost.

**Table 3:** Training and cross-validation accuracy of the SVM classification for ATR-FTIR spectral data.

Plant Parts	Training Accuracy	Cross-Validation Accuracy
All parts	84.62	61.54
Root	100	77.78
Stem	88.84	44.44
Leaf	100	33.33

Note: Data are presented as the mean value of various plant parts of the *C. arietinum* samples grown in 3 conditions: (1) Au NPs, (2) CNTs, and (3) control group. The results demonstrate the possibility of automatic classification of plants based on nanoparticle-inclusion in plant samples using PCA and linear SVM with accuracy of nearly 85%.

## Data Availability

Data generated in this article are available upon request from the authors. Authors’ e-mail addresses are provided as: F. Candan at feyzacandan2002@yahoo.com; Y. Markushin at ymarkushin@desu.edu; and G. Ozbay at gozbay@desu.edu.
